# Zileuton protects against arachidonic acid/5-lipoxygenase/leukotriene axis-mediated neuroinflammation in experimental traumatic brain injury

**DOI:** 10.3389/fphar.2025.1516836

**Published:** 2025-06-05

**Authors:** Xiaofeng Xu, Yan Wang, Xiaomei Li, Yang Yang, Dianxu Yang, Wenqi Tang, Jin Lu, Fang Yuan

**Affiliations:** ^1^ Department of Neurology, Shanghai Sixth People’s Hospital Affiliated to Shanghai Jiao Tong University School of Medicine, Shanghai, China; ^2^ Department of Neurosurgery, Xuzhou First People’s Hospital Municipal Hospital of Xuzhou Medical University, Xuzhou, Jiangsu, China; ^3^ Department of Nephrology, Shanghai Sixth People’s Hospital Affiliated to Shanghai Jiao Tong University School of Medicine, Shanghai, China; ^4^ Department of Neurosurgery, Shanghai Sixth People’s Hospital Affiliated to Shanghai Jiao Tong University School of Medicine, Shanghai, China; ^5^ Department of Critical Care Medicine, Shanghai Sixth People’s Hospital Affiliated to Shanghai Jiao Tong University School of Medicine, Shanghai, China; ^6^ Department of Pharmacy, Shanghai Sixth People’s Hospital Affiliated to Shanghai Jiao Tong University School of Medicine, Shanghai, China

**Keywords:** zileuton, 5-lipoxygenase, leukotriene, microglial cells, neuroinflammation, traumatic brain injury

## Abstract

**Introduction:**

Traumatic brain injury (TBI) is a leading cause of death and disability globally. Several studies have shown that 5-lipoxygenase (5-LOX) inhibition reduces leukotriene (LT) release and the inflammatory response, attenuating the development of respiratory diseases, myocardial infarction, and ischemic cerebral injury. However, its role in the pathophysiology of TBI remains unclear.

**Methods:**

Controlled cortical impact injury was induced to construct a mouse model of TBI. Pericontusional brain tissue samples from sham and TBI mice at 7 days after injury were used for RNA-seq analysis. Altered gene enrichment following TBI, based on Kyoto Encyclopedia of Genes and Genomes (KEGG) pathway analysis, was quantified through real-time polymerase chain reaction (RT-PCR). Immunocytochemistry, Western blotting, and single-cell sequencing experiments were also performed to analyze 5-Lox protein expression. Arachidonic acid (AA) was detected through liquid chromatography mass spectrometry/mass spectrometry. Enzyme-linked immunosorbent assay was used to detect LTB4 release after TBI with or without zileuton treatment. Brain damage, blood–brain barrier disruption, and neuronal apoptosis were detected through histological examination. Neurological outcomes were determined through rotarod and fear conditioning tests.

**Results:**

TBI induced significant upregulation of genes related to the AA metabolic pathway, particularly the AA/5-LOX/LT axis, as verified by RT-PCR. AA and LTB4 production increased significantly after TBI. The expression levels of Pla2g4a, which hydrolyses phospholipids to release AA, and 5-Lox, which in turn act downstream to convert AA to LT, were dramatically upregulated up to 7 days after TBI. 5-LOX accumulated in the cytoplasm of activated ameboid microglial cells. In vivo, 5-LOX inhibition with zileuton blocked LT release and reduced microglial activation and the production of inflammatory cytokines, including Il-1β, Ccl7, Spp1, Ccr1, Ccl2, and Il-10. Zileuton also reduced TBI-induced lipid ROS and neuronal cell apoptosis, ameliorating brain damage compared to the vehicle group and improving neurological outcomes after TBI. Mechanically, TBI-induced LT upregulation may stimulate BV2 microglial activation through the ERK, NF-κB, and Akt pathways.

**Conclusion:**

Our findings demonstrated the role of 5-LOX in TBI and its potential as a therapeutic target in TBI treatment.

## Introduction

Traumatic brain injury (TBI) is a leading cause of death and disability globally, imposing a significant economic burden on societies and families (Robba et al., 2024; Maas et al., 2017). Direct physical assault affecting brain tissue sets off a secondary cascade of damage marked by metabolic dysregulation, oxidative stress, neuroinflammation, and neuronal cell death (Zhang et al., 2024; Yang et al., 2019). An understanding of the pathological processes that occur following TBI is crucial for the development of novel therapies to mitigate potential damage.

TBI initiates acute inflammation, leading to immune cell infiltration and subsequently progressing to chronic inflammation with persistent microglial activation (Witcher et al., 2021; Wangler and Godbout, 2023). Arachidonic acid (AA) metabolism contributes to several neuroinflammatory diseases, including TBI (Gorica and Calderone, 2022). In the early stages of TBI, AA is released from inner membrane phospholipids by phospholipase enzymes (Chen, 2023). Lipoxygenases (LOX), particularly 5-lipoxygenase (5-LOX), play an important role in further metabolizing AA. The resulting proinflammatory leukotrienes (LTs) recruit neutrophils and monocytes to injury sites, facilitating debris clearance and cytokine secretion (Alam et al., 2020). While this acute inflammatory response is crucial for brain tissue repair and clearing debris, it also increases the risk of secondary neuronal injury. In later neuroinflammation stages, AA-derived lipid mediators shift from proinflammatory to proresolving, including resolvins and prostaglandins (Sansbury and Spite, 2016). This transition halts immune cell infiltration and modulates microglial activation to resolve the inflammatory process (Gorica and Calderone, 2022). Manipulating microglial activity and inflammatory responses by regulating AA metabolism is a promising therapeutic approach for TBI and neurodegenerative diseases with neuroinflammatory involvement.

5-LOX, a rate-limiting enzyme in proinflammatory LT production (Michael et al., 2020), interacts with 5-LOX-activating protein (FLAP) to metabolize AA into 5(S)-hydroperoxide (5-HPETE). This is then converted to leukotriene A4 (LTA4), the precursor of LTB4 and cysteinyl LTs (LTC4, LTD4, and LTE4). These LTs can compromise blood–brain barrier integrity, contributing to neuroinflammation and brain edema in experimental ischemia cerebral injury and TBI (Yan et al., 2021; Corser-Jensen et al., 2014). Inhibition of 5-LOX reduces LT production, significantly diminishing the size of focal cerebral infarction induced by middle cerebral artery occlusion (Yan et al., 2021). Blocking LT synthesis through FLAP inhibition also alleviates TBI and promotes neurobehavioral recovery (Corser-Jensen et al., 2014).

Zileuton, a selective 5-LOX inhibitor, effectively reduces inflammatory components and cellular infiltration by inhibiting LT production in inflammation-related diseases. In addition to its role in respiratory diseases, such as asthma, obstructive pulmonary disease, and pulmonary hypertension (Drago et al., 2021), zileuton exhibits diverse protective effects. Mechanistically, it inhibits myocardial infarction-induced NF-κB expression and reduces cardiomyocyte apoptosis (Abueid et al., 2017). Moreover, it attenuates inflammatory cytokine release and alleviates ischemic brain injury by inhibiting the PI3K/Akt pathway (Tu et al., 2016).

However, little is known about the involvement of 5-LOX in TBI pathophysiology. First, the modification of AA metabolism through the 5-LOX axis following TBI remains poorly understood. Second, controversy persists regarding the cellular localization of 5-LOX. It has been identified in the cytosol and nucleus of neutrophils, neurons, GFAP-positive glial cells, and endothelial cells (Yan et al., 2021). The temporal and spatial expression of 5-LOX following TBI remains unknown. Third, the cell-specific distribution of 5-LOX is significant for its functional performance. Investigating whether TBI-induced upregulation of 5-LOX and subsequent release of LTs modulate the inflammatory response after TBI requires further exploration. Finally, evaluating the impact of 5-LOX inhibition on TBI-induced brain damage and neurological deficits will be instrumental in advancing novel TBI therapies.

In this study, we conducted an RNA-seq analysis of pericontusional brain tissue samples from sham and TBI mice at 7 days post-injury. We observed substantial enrichment of genes associated with the AA pathway, particularly the AA/5-LOX/LT axis; such genes were upregulated after TBI. The production of AA and LTs significantly increased post-TBI. The expression of Pla2g4a, responsible for hydrolyzing phospholipids to AA, and of downstream 5-LOX, which converts AA to LTs, was significantly upregulated up to 7 days after TBI. Importantly, 5-LOX expression specifically increased in the cytoplasm of activated ameboid microglial cells. Furthermore, inhibiting 5-LOX with zileuton effectively blocked LT release, and reduced microglial activation and inflammatory cytokine production, resulting in less brain damage compared to the vehicle group and improved neurological outcomes after TBI. Taken together, our findings contribute to the growing body of evidence supporting inhibition of 5-LOX activity by zileuton, demonstrate a role for 5-LOX in TBI, and highlight its potential as a therapeutic target for TBI treatment.

## Materials and methods

### Animals and TBI models

Adult male C57BL/6J mice (20–25 g) were purchased from Shanghai JieSiJie Laboratory Animal Co.,Ltd. and housed under pathogen-free conditions. Mice in drug treatment group received intraperitoneal injection of Zileuton (CSN17529, CSN pharm, dissolved in corn oil) at 10 mg per kg body weight per day after TBI. These doses have been found to significantly inhibit the activity of 5-LOX and be beneficial to alleviate the inflammatory response of pleurisy and antigen-induced responses (Rossi et al., 2010; Scuri et al., 1998). The first injection was performed 30 min after TBI induction. All animal-related experimental procedures were performed in accordance with the National Institutes of Health guidelines and approved by the Laboratory Animal Ethical Committee of Shanghai Jiaotong University.

A controlled cortical impact (CCI) induced TBI model (PinPoint Precision Cortical Impactor PCI3000; Hatteras Instruments Inc., Cary, NC, United States) was performed as previously described (Wang et al., 2022; Yuan et al., 2016). The injury severity parameters were 3-mm diameter impactor tip, velocity of 1.5 m/s, deformation depth of 1.5 mm, and dwell time of 100 ms. Sham animals underwent exactly the same procedure as the injured mice, but the impact was not performed.

### RNA-sequencing

Whole-genome gene expression analysis was performed using the brain tissue from sham, TBI and Zileuton treated mice (n = 3 per group) at 7 days after TBI. The total RNA was extracted using Trizol, and cDNA samples were sequenced by Shanghai Applied Protein Technology Co. Ltd. All the differentially expressed genes (bilateral *t*-test p < 0.05) were used for Kyoto Encyclopedia of Genes and Genomes (KEGG) pathway enrichment analysis. Enriched pathways after TBI without zileuton treatment were in Supplementary Additional File 1 and with zileuton treatment were in Supplementary Additional File 2.

### Real-time polymerase chain reaction

Total RNA from mouse brains was extracted using Trizol, and reverse transcribed using TransScript One-Step gDNA Removal and cDNA Synthesis SuperMix (TransGen Biotech, China). The resulting cDNAs were used for PCR using the TB Green^®^ Premix Ex Taq™ (Takara). Relative abundance of mRNA was determined on a 7,500 real-time PCR system (Thermo Fisher Scientific). Fold changes of RNA levels were calculated using the ΔΔCt method and were analysed by Student’s t-test. The primer pair sequences used for RT-PCR reactions for *in vivo* studies are listed in Supplementary Additional File 3.

### Western blot assay

Primary antibodies used for Western blot analyses include rabbit anti-Pla2g4a (1:1,000; #A10270, Abclonal, Wuhan, China), rabbit anti-5 Lipoxygenase (1:500; #10021-1-Ig, Proteintech), rabbit anti-5 Lipoxygenase (1:500; #ab169755, Abcam), rabbit anti-Erk1/2 (1:1,000, #4695, Cell Signaling Technology, Beverly, MA, United States), rabbit anti-Phospho-Erk1/2 (1:1,000, #4370, Cell Signaling Technology), rabbit anti-Phospho-Akt (S473) (1:1,000, #4060, Cell Signaling Technology), rabbit anti-Phospho-Akt (T308) (1:1,000, #13038, Cell Signaling Technology), rabbit anti- Akt (1:1,000, #4691, Cell Signaling Technology), Rabbit anti-Phospho-NF-κB p65 (1:1,000, #3033, Cell Signaling Technology), rabbit anti- NF-κB p65 (1:1,000, #8242, Cell Signaling Technology), and rabbit anti- GAPDH (1:1,000, #5174, Cell Signaling Technology).

### Liquid chromatography mass spectrometry/mass spectrometry based metabolic analysis

Mice were anesthetized and perfused transcardially with chilled phosphate-buffered saline (PBS) (n = 11–12 per group). Brains were removed and maintained at −80°C until metabolic analysis. The extraction of metabolites and LC-MS/MS-based metabolic analysis were performed by Shanghai Lu-Ming Biotech Co., Ltd. Differential metabolites were selected with variable importance of projection values greater than 1.0 and p-values less than 0.05.

### Enzyme linked immunosorbent assay

Brain tissues were collected on day 3 after TBI with or without Zileuton treatment, and stored at the −80°C before use. We carried out an enzyme immunoassay to measure the LTB4 levels in tissues using an ELISA kit (#520111, Cayman) following the manufacturer’s instruction.

### Immunocytochemistry and image analysis BODIPY staining

Free floating brain sections (30-μm thick) were fixed with 4% paraformaldehyde, blocked with 10% normal goat serum, and incubated overnight at 4°C with the following primary antibodies: rabbit anti-5 Lipoxygenase (1:100; #ab169755, Abcam) and rat anti-CD68 (1:1,000, #MCA 1957, Bio-RAD, United States). After washing in phosphate-buffered saline, sections were incubated with Alexa-488- or Alexa-594-conjugated secondary antibodies (1:500 dilution; Life Technologies). For BODIPY staining, sections were washed once in PBS and incubated in PBS with C11-BODIPY 581/591 (1:2000 from a 1 mg/mL stock solution in dimethylsulfoxide (DMSO); Life Technologies). Nuclei were stained with 4′6-diamidino-2-phenylindole (DAPI, 1:5000; Sigma). Photographs were taken with a confocal microscope (Leica, Solms, Germany) for further analyses.

### Lesion volume assessment

To determine the lesion volume after TBI, serial sections (30-μm thick) of brain were collected from −0.5 mm to −3.5 mm posterior to bregma. The first of every ten consecutive sections was selected for staining with cresyl violet (Beyotime, Nantong, China). The areas of impact defects were measured using ImageJ software (National Institutes of Health, Bethesda, MD, United States). Lesion volume was calculated by multiplying the sum of all defects by interval distance.

### Neurobehavioral tests

The Rotarod test was used to evaluate motor deficits. All mice were trained on the rotarod for 3 consecutive days before the induction of CCI. The average time for mice to fall was recorded on days 7 after TBI.

Fear conditioning was used to assess cognitive function. The test was conducted with the ANY-maze fear conditioning system (Stoelting Co.). Briefly, during the training phase, 1 day before TBI induction, mice were placed into the conditioning chamber and habituated to their surroundings for 2 min. Following habituation, the mice received three pairings between a tone/light (20 s) and an electrical shock (1 s, 0.5 mA). The inter-trial interval between each of the pairings is 2 min. During the test phase, the procedure and context were identical to those used on training phase, except that the shock is not presented. The chamber is cleaned with 70% ethanol after each mouse.

### Terminal dexynucleotidyl transferase mediated dUTP nick end labeling (TUNEL) assay

Apoptotic cells were detected by a commercial One Step TUNEL Apoptosis Assay kit (Beyotime, Nantong, China) following manufacturer’s instruction. Briefly, brain sections were subjected to react with the TUNEL mixture solution for 1 h at 37°C, and then stained with DAPI. Apoptotic cells were observed and recorded under a confocal fluorescence microscope.

### Cell culture and reagents

BV2 microglial cells were provided by the China Center for Type Culture Collection (CCTCC, Wuhan, Hubei, China), HT22 hippocampal neuronal cells were purchased from American Type Culture Collection (ATCC, Manassas, VA, United States). All the cells were cultured in complete Dulbecco’s modified Eagle medium (DMEM) supplemented with 10% heat-inactivated fetal bovine serum (FBS) and 100 U/mL penicillin/streptomycin. LTB4 (s42020826, Absin), LTE4 (ab141690, Abcam), RSL3 (HY-100218A, MedChemExpress, MCE) and Erastin (HY-15763, MCE) were purchased commercially.

### Statistical analysis

Statistical analyses and graphic representation were performed using GraphPad Prism 9.0 (GraphPad Software, San Diego, CA, United States). All data from triplicated independent experiments are represent as mean ± SD. The equality of variance was assessed by Levene’s test between the compared groups. The p values were calculated with two-tailed unpaired Student’s t-test, one-way ANOVA with Dunnett’s or Tukey’s multiple comparisons test as indicated in corresponding figure legends p < 0.05 were considered statistically significant.

## Results

### AA metabolism was upregulated after experimental TBI

**FIGURE 1:**
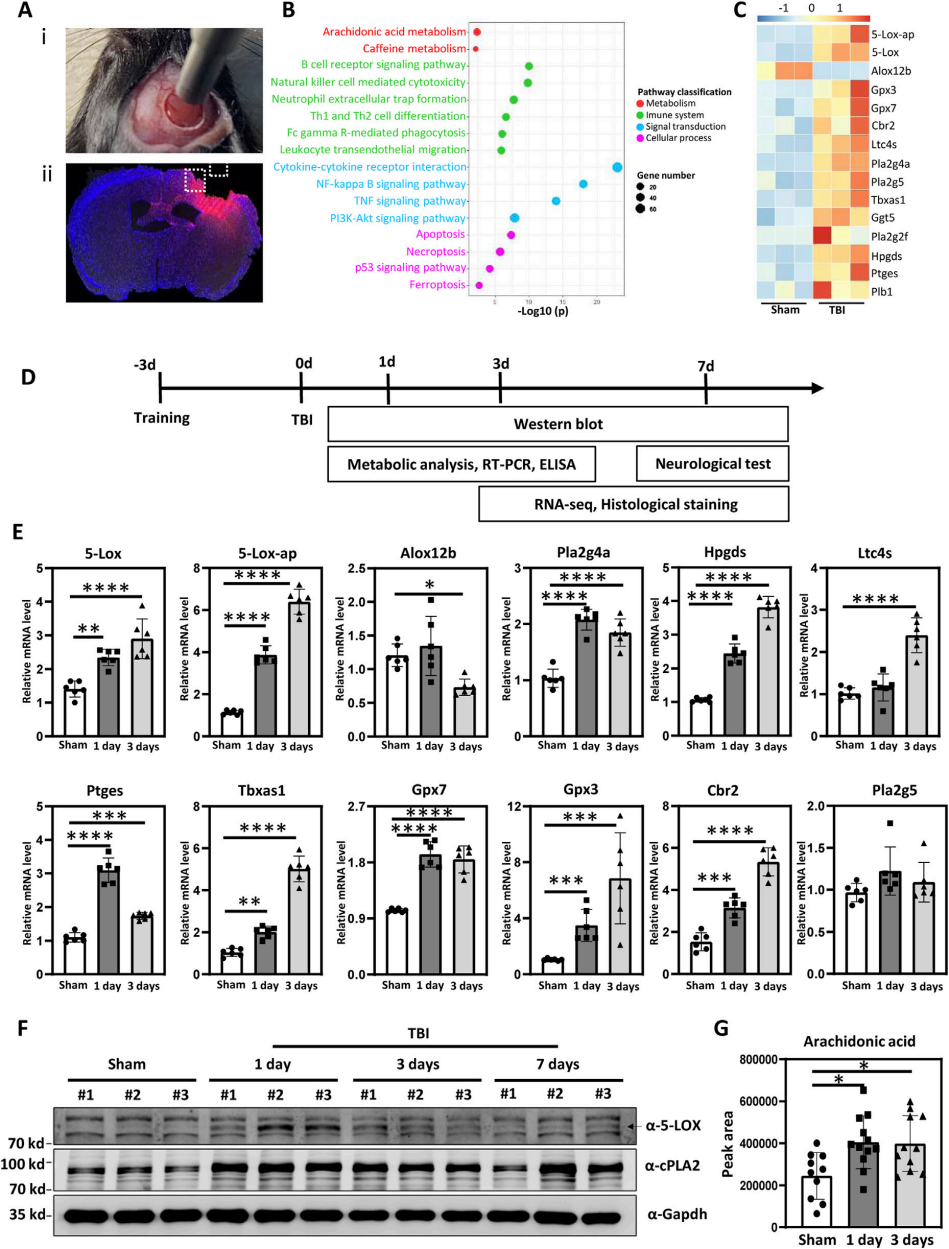
Arachidonic acid (AA) metabolism was upregulated after experimental traumatic brain injury (TBI). [**(A)**i] The process of controlled cortical impact (CCI) in mice. [**(A)**ii] Dashed box illustrates the pericontusional region of interest after TBI. **(B)** Kyoto Encyclopedia of Genes and Genomes (KEGG) pathway enrichment analysis of the genes upregulated after TBI. Significantly enriched metabolic, immune system, signal transduction, and cellular process pathways are shown. **(C)** Heat map showing differentially-expressed genes related to AA metabolism in the TBI-induced pericontusional brain tissue at 7 days based on RNA-seq analysis (n = 3 for each group). **(D)** Schematic overview of the experimental design. Mice were subjected to TBI using the CCI model, and the resulting damage phenotypes were analyzed at indicated time points post-injury. **(E)** mRNA levels of 5-Lox, 5-Lox-ap, Gpx3, Gpx7, Cbr2, Pla2g4a, Ltc4s, Tbxas1, Hpgds, and Ptges were measured in sham and TBI mice at 1 and 3 days after TBI (n = 6 for each group, *p < 0.05, **p < 0.01, ***p < 0.001, ****p < 0.0001). **(F)** Time course of 5-LOX and Pla2g4a/cPLA2 protein expression in pericontusional tissue. **(G)** AA concentrations in brain tissue were measured in sham and TBI mice at 1 and 3 days after TBI (n = 10, = 12, and = 11 for the sham, 1-day, and 3-day groups, respectively, *p < 0.05).

To investigate potential mechanisms underlying TBI, we used a mouse model of controlled cortical impact (CCI) (Figure 1A). RNA-seq and subsequent pathway enrichment analysis using the Kyoto Encyclopedia of Genes and Genomes (KEGG) based on the upregulated genes, revealing distinct changes in the AA pathway following TBI (Figure 1B). The heat map showed significant changes in genes associated with AA metabolism, including 5-Lox-ap, 5-Lox, Alox12b, Gpx3, Gpx7, Cbr2, Ltc4s, Pla2g4a, Pla2g5, Tbxas1, Ggt5, Pla2g2f, Hpgds, Ptges, and Plb1 (Figure 1C). The subsequent experimental design is shown in Figure 1D. Validation of the RNA-seq results through RT-PCR revealed significant upregulation of AA-related genes, including 5-Lox, 5-Lox-ap, Gpx3, Gpx7, Cbr2, Pla2g4a, Ltc4s, Tbxas1, Hpgds, and Ptges, following TBI (Figure 1E). These findings highlight the impact of AA/5-LOX during CCI-induced brain injury in mice.

Pla2g4a, a member of the cytosolic phospholipase A2 family, hydrolyzes inner membrane phospholipids to release AA (Gorica and Calderone, 2022; Sansbury and Spite, 2016), which is subsequently metabolized by LOX to produce LTs and eicosatrienoic acids (Wang et al., 2021). Our Western blot analysis revealed a substantial increase in Pla2g4a and downstream 5-LOX expression at 1 day post-TBI, followed by a slight decrease at 3 days. Elevated levels were maintained for up to 7 days. The expression pattern of 5-LOX mirrored that of Pla2g4a/cPLA2 (Figure 1F). To confirm the functional impact of cPLA2 upregulation, AA levels were assessed in sham and injured mouse brain tissues using liquid chromatography-mass spectrometry. Compared to the sham group, AA levels were significantly elevated by approximately 1.5-fold in the TBI group at both 1 and 3 days after injury (Figure 1G). These results indicate that TBI induces the upregulation of cPLA2 and its product, AA, leading to increased downstream 5-LOX expression and LT production.

### Upregulated 5-LOX expression in activated microglia after TBI

**FIGURE 2:**
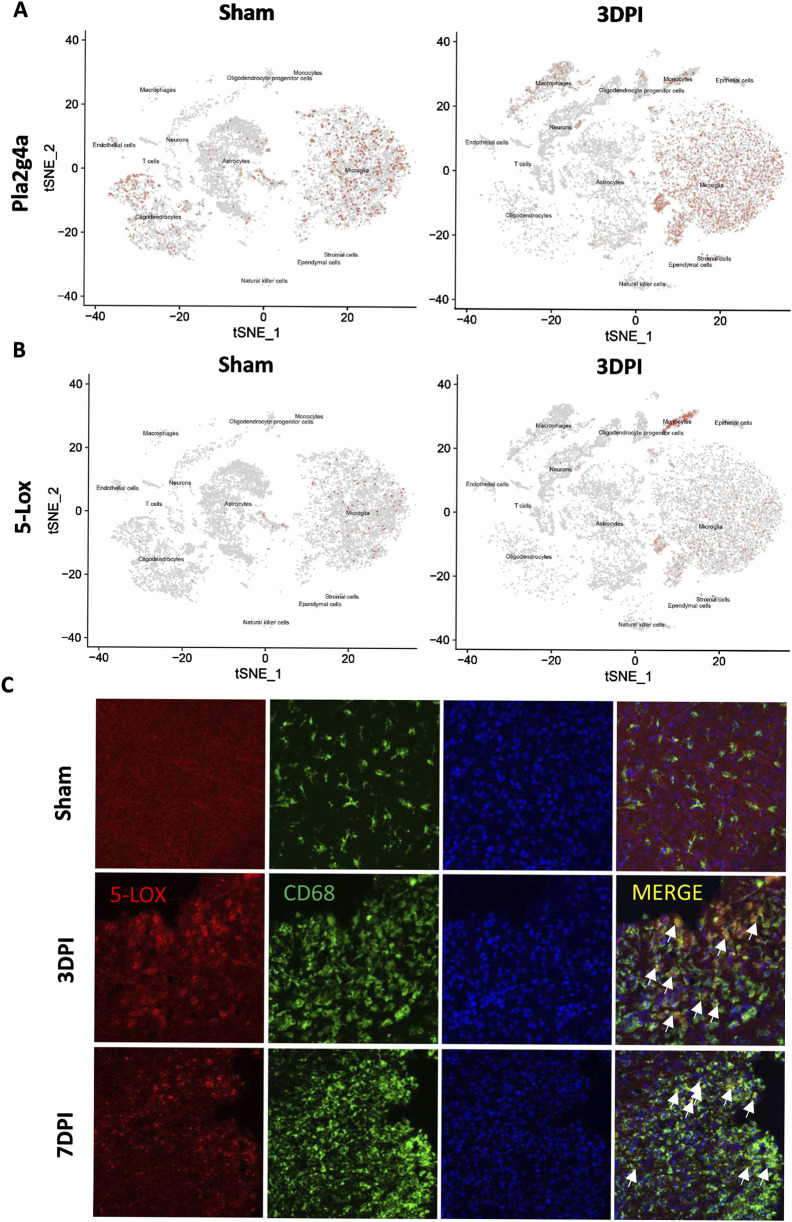
The expression of 5-LOX was upregulated in activated microglia/monocytes after TBI. **(A, B)** tSNE maps of mouse brain cells showing cell type-specific expression of Pla2g4a and 5-Lox in sham and TBI mice at 3 days after TBI. **(C)** Representative 5-LOX/CD68 immunofluorescence staining in the brains of sham and TBI mice at 3 and 7 days after TBI. White arrows indicate 5-LOX/CD68 co-localized inflammatory cells (scale bar = 10 μm).

The enzyme 5-LOX plays a crucial role in AA metabolism, which results in LT production (Wu et al., 2019). To investigate the potential role of 5-LOX in the pathological progression of TBI, we examined single-cell RNA-seq data from TBI mice for cell type-specific expression patterns of Pla2g4a and 5-Lox. Pla2g4a was predominantly expressed in microglia and oligodendrocytes, being significantly upregulated in macrophages, monocytes, and microglia (Figure 2A). Meanwhile, 5-Lox expression remained low in the sham group but significantly increased in microglia and monocytes following TBI (Figure 2B).

Immunofluorescence staining of CD68/5-LOX was performed to validate these findings, confirming that 5-LOX expression was markedly upregulated in the cytoplasm of activated ameboid CD68^+^ inflammatory cells without significant changes in NeuN^+^ neurons or GFAP^+^ astrocytes (Figure 2C). The cell type-specific upregulation of 5-LOX associated with microglial activation prompted us to explore the effects of 5-LOX inhibition on LT biosynthesis suppression and the inflammatory response after TBI.

### Zileuton reduced TBI induced brain damage and neurological deficits

**FIGURE 3:**
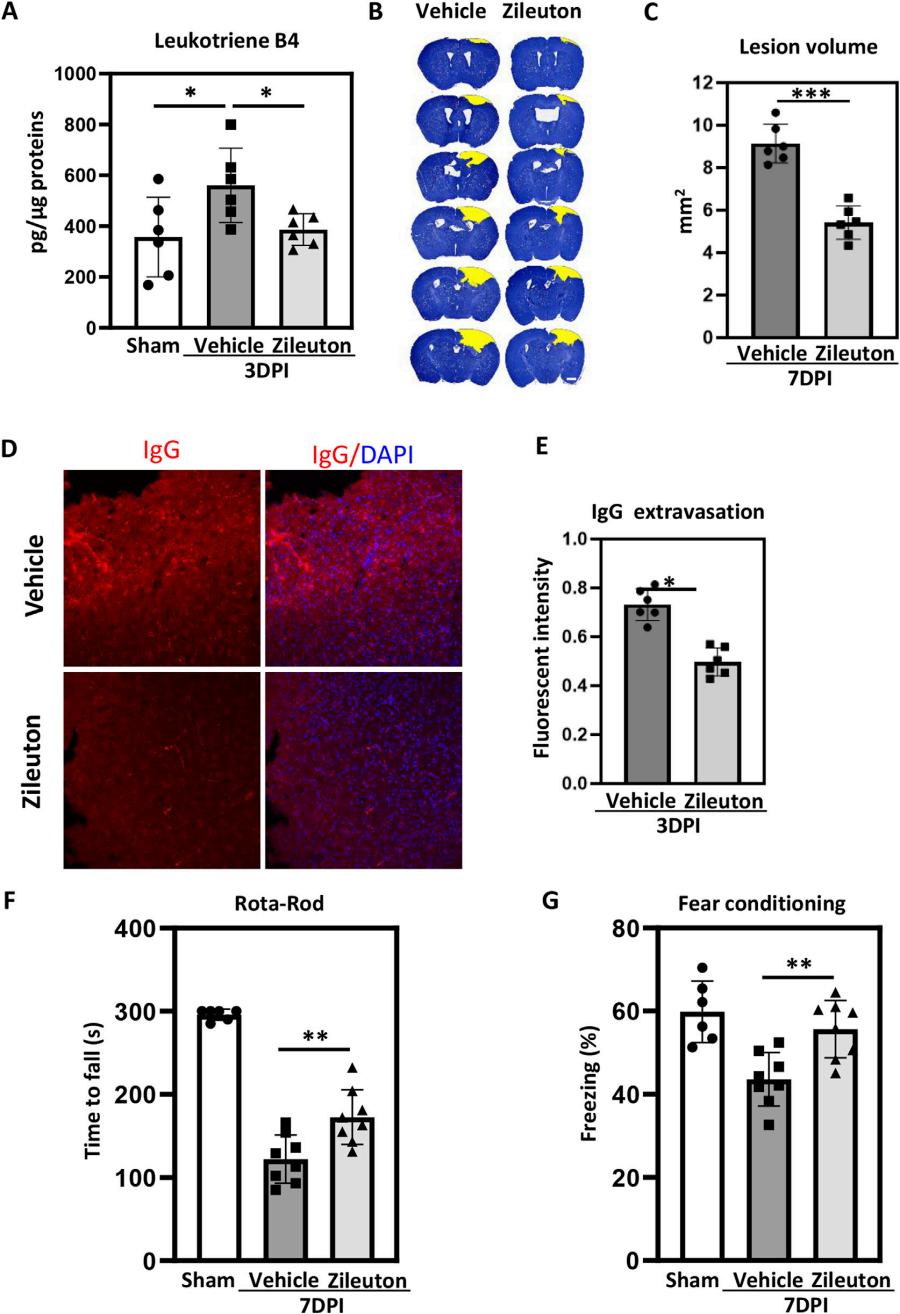
Zileuton reduced TBI-induced brain damage and neurological deficits. **(A)** Leukotriene B4 levels in brain tissue were measured in sham, TBI, and zileuton-treated mice at 3 days after TBI (n = 6 for each group, *p < 0.05, **p < 0.01). **(B)** Representative cresyl violet-stained brain sections from the vehicle- and zileuton-treated groups at 7 days after TBI. Lesions are outlined in yellow (scale bar = 10 μm). **(C)** Lesion volume was quantified and reported as mean ± SEM. **(D)** Representative IgG immunostaining of brain sections from vehicle- and zileuton-treated groups at 3 days after TBI (scale bar = 10 μm). **(E)** Quantification of fluorescence intensity for IgG extravasation in vehicle- and zileuton-treated groups 3 days after TBI (n = 6 for each group, *p < 0.05). **(F)** Rotarod test in vehicle- and zileuton-treated groups at 7 days after TBI (n = 6–8 mice/group) **(G)** Fear conditioning test; percentage of freezing time during the 5-minute test at 7 days after TBI (n = 6–8 mice/group, *p < 0.05).

Zileuton, a selective 5-LOX inhibitor, has demonstrated efficacy in alleviating various inflammation-related diseases by suppressing LT formation (Peters-Golden and Henderson, 2007). Therefore, we investigated whether 5-LOX inhibition in the TBI model could influence LT production in addition to mitigating TBI. ELISA was used to quantify LTB4 levels in pericontusional tissue at 3 days after TBI. LTB4 production increased significantly after TBI, and zileuton (Figure 3A) administration completely blocked this increase at 3 days after injury, confirming the inhibitory effect of zileuton on 5-LOX.

Subsequently, serial brain sections were used to assess the impact of 5-LOX inhibition on TBI-induced pathological changes in both the vehicle and zileuton groups. Cresyl violet staining indicated substantial defects in the ipsilateral brain parenchyma following CCI, with zileuton treatment markedly reducing lesion volume at 7 days post-TBI (Figure 3B). The mean lesion volume in the vehicle group was 9.54 ± 0.65 mm^3^, and the zileuton group exhibited a significantly reduced contusion volume compared to the vehicle group (*p* < 0.001; Figure 3C). Additionally, IgG staining was performed to assess the integrity of the blood–brain barrier after TBI. CCI induced significant IgG extravasation in the ipsilateral cortex (Figures 3C,D), and zileuton administration significantly reduced IgG extravasation at 3 days after injury (*p* < 0.05; Figure 3E).

To explore the effects of 5-LOX inhibition on TBI-induced neurological deficits, the rotarod and fear conditioning tests were conducted in both vehicle- and zileuton-treated mice. Compared to the vehicle group, the zileuton-treated group exhibited significant improvements in motor function at 7 days after TBI (*p* < 0.05; Figure 3F). Similarly, the fear conditioning test revealed significant enhancement in memory function in the zileuton treatment group at 7 days post-TBI compared to the vehicle group (*p* < 0.05; Figure 3G). Taken together, these results demonstrated that 5-LOX inhibition with zileuton ameliorates TBI-induced brain damage and neurological deficits.

### Zileuton reduced TBI-induced microglial activation

**FIGURE 4:**
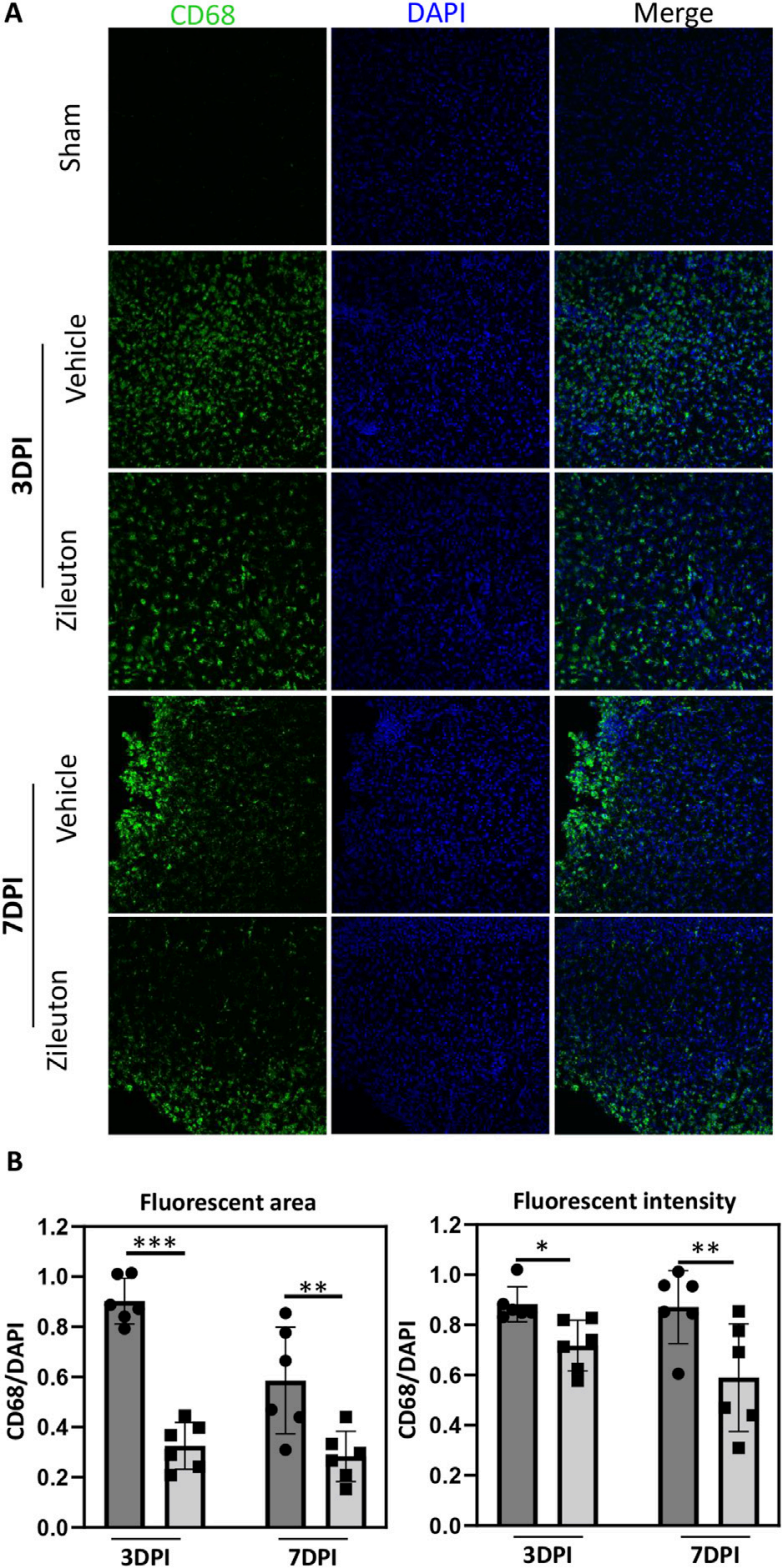
Zileuton reduced TBI induced microglia/macrophage activation. **(A)** Immunofluorescence staining for CD68 in the pericontusional regions of TBI and zileuton-treated mice at 3 and 7 days after TBI (Scale bar = 10 μm). **(B)** Quantification of CD68 fluorescence area and intensity relative to 4′6-diamidino-2-phenylindole (DAPI, n = 6 for each group, *p < 0.05).

We further investigated the impact of 5-LOX inhibition on microglial activation in the TBI model. Microglial activation was assessed through CD68 immunofluorescence staining in mice treated with daily injections of the 5-LOX inhibitor zileuton. In the sham group, no significant microglial activation was expected. In the vehicle group, extensive CD68 immunolabeling was observed in pericontusional regions of the ipsilateral hemisphere at both 3 and 7 days post-TBI. Intraperitoneal zileuton administration appeared to reduce TBI-induced microglial activation in the corresponding area (Figure 4A). Statistical analysis of CD68 in mouse brain sections revealed that zileuton treatment significantly decreased both the area and intensity of fluorescence following TBI (Figure 4B).

### 5-LOX inhibition decreased the TBI-induced expression of inflammatory cytokines

**FIGURE 5:**
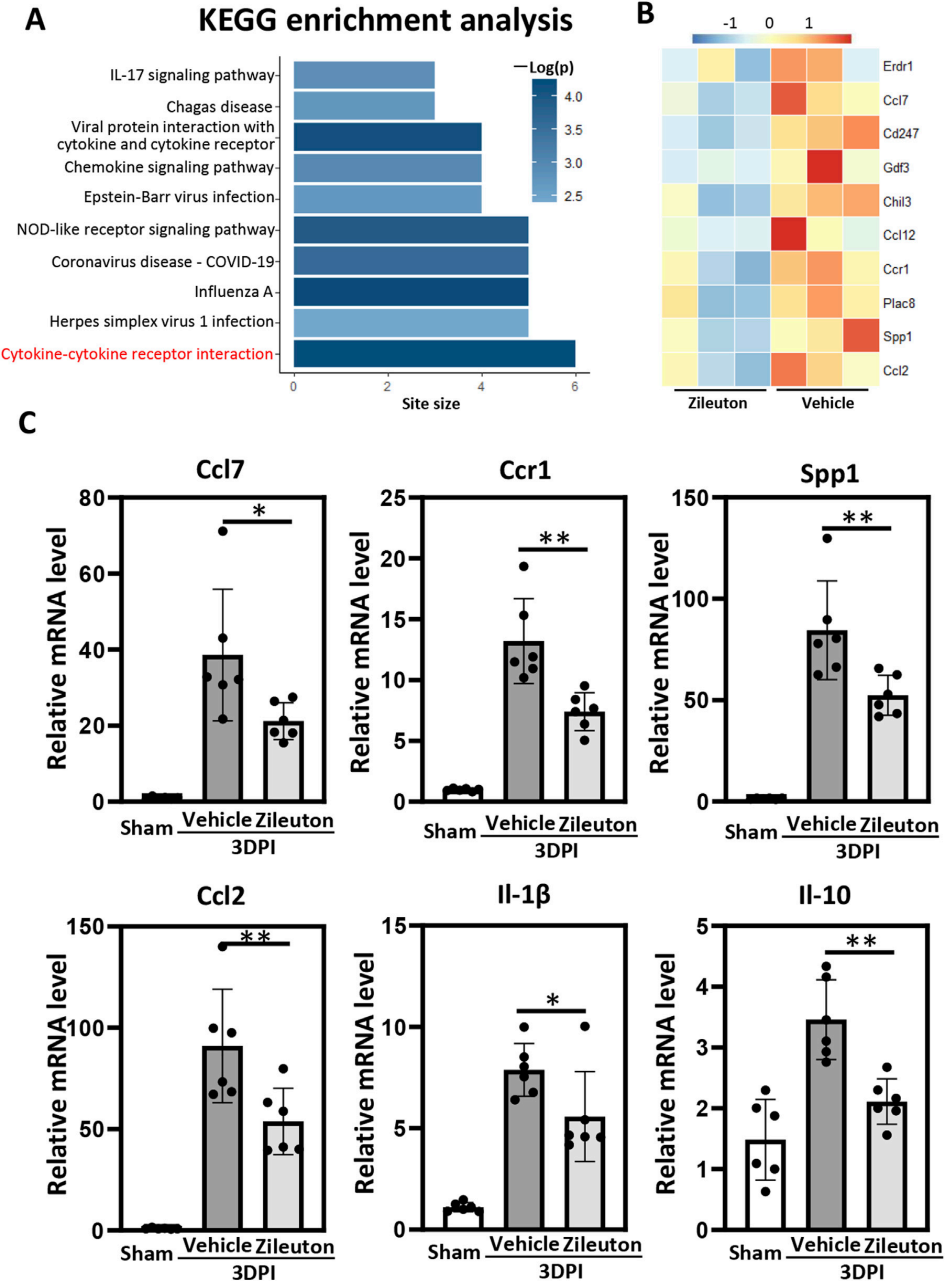
**(A)** Kyoto Encyclopedia of Genes and Genomes (KEGG) pathway enrichment analysis of the genes downregulated by zileuton treatment. The top most 10 significantly enriched pathways (*p* < 0.05 by Fisher’s exact test) are shown. **(B)** Heat map showing differentially expressed genes in the pericontusional brain tissue between the vehicle- and zileuton-treated groups at 7 days after TBI based on RNA-seq analysis (n = 3 for each group). **(C)** mRNA levels of ccl7, ccr1, ssp1, ccl2, il1β, and il10 were measured in the vehicle- and zileuton-treated mice at 3 days after TBI (n = 6 for each group, *p < 0.05, **p < 0.01).

To investigate the potential mechanisms underlying 5-LOX inhibition in TBI pathogenesis, we conducted RNA-seq analysis of brain tissue samples from pericontusional regions in both the vehicle- and zileuton-treated groups at 7 days post-TBI. A total of 136 genes exhibited significant differences between the vehicle- and zileuton-treated mice, with 52 genes upregulated and 84 downregulated by zileuton after injury. Subsequently, KEGG pathway enrichment analysis based on the downregulated genes revealed that the most significant effect was on the cytokine–cytokine receptor interaction pathway, followed by pathways associated with the inflammatory response (Figure 5A). The heat map shows the top 10 genes most significantly downregulated by zileuton administration, including Erdr1, Ccl7, Cd247, Gdf3, Chil3, Ccl12, Ccr1, Plac8, Spp1, and Ccl2 (Figure 5B).

The neuroinflammatory response following TBI correlated positively with injury severity. We validated the mRNA levels of inflammatory cytokines and cytokine receptors from the RNA-seq results, as well as the levels of the cytokines TNF-α, Il-17, Il-6, Il-1β, and Il-10, in pericontusional regions using quantitative polymerase chain reaction (qPCR). Our findings demonstrated significant upregulation of inflammation-related genes, including Ccl7, Ccl12, Ccr1, Spp1, Ccl2, Il-10, TNF-α, Il-17, Il-6, and Il-1β, at 3 days after injury. Relative mRNA levels of Il-1β, Ccl7, Spp1, Ccr1, Ccl2, and Il-10 were significantly decreased in the zileuton-treated TBI group compared to the vehicle group (Figure 5C). However, no significant decrease in TNF-α, Il-17, Ccl12, or Il-6 was observed in the zileuton-treated group (Supplementary Figure S1). In conclusion, these results indicate that 5-LOX inhibition ameliorates neuroinflammation by reducing proinflammatory cytokine release and downregulating cytokine–cytokine receptor interaction.

### 5-LOX inhibition reduced TBI induced lipid ROS and neuronal cell apoptosis

**FIGURE 6:**
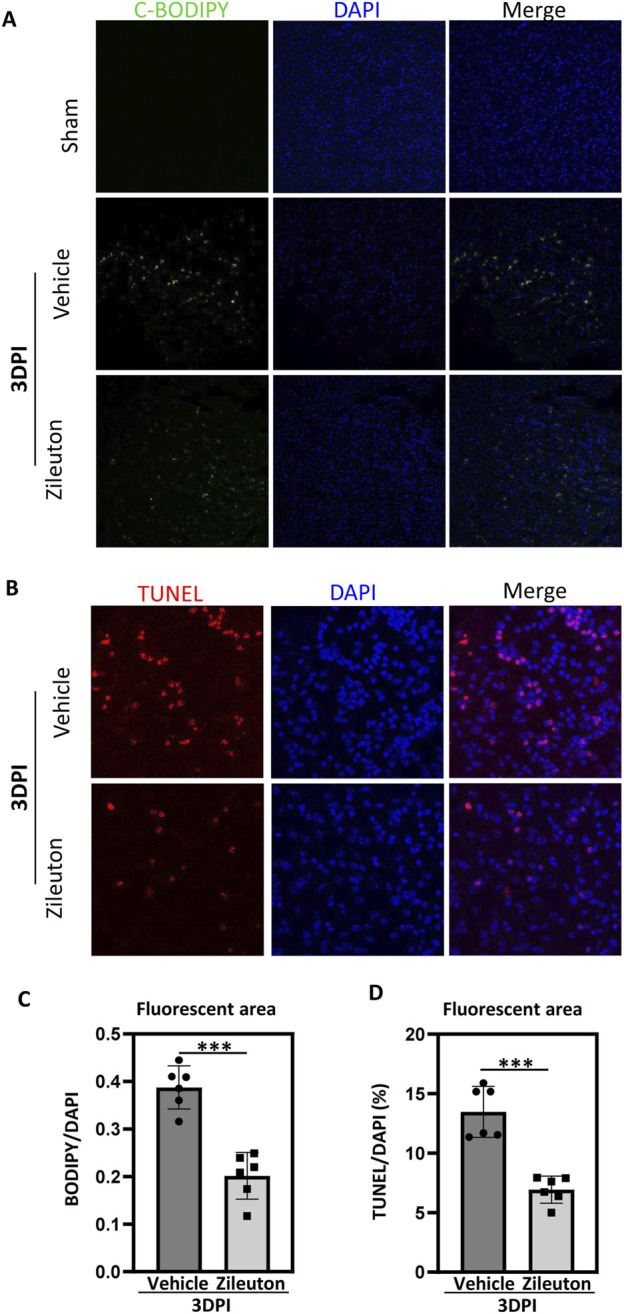
5-LOX inhibition reduced TBI-induced lipid ROS generation and neuronal cell apoptosis. **(A)** Representative confocal images of brain sections labeled with C11-BODIPY and DAPI from vehicle- and zileuton-treated mice at 3 days after TBI. Green and blue colors indicate lipid ROS (peroxidized lipids) and the nucleus, respectively. **(B)** Apoptotic cells in brain tissue sections were detected through TUNEL staining. **(C)** Quantification of the area of C11-BODIPY/DAPI fluorescence in **(A)**. **(D)** Quantification of the area of TUNEL/DAPI fluorescence in **(B)**. (n = 6 for each group, *p < 0.05, **p < 0.01).

Accumulation of lipid peroxidation is known to play a pivotal role in secondary injury following TBI, contributing to the inflammatory response and programmed cell death (Tarudji et al., 2023). Therefore, we used the fluorescent probe C11-BODIPY (581/591) to detect lipid ROS accumulation after TBI, with or without zileuton treatment. Control mouse brains showed no lipid ROS, while significant accumulation of lipid ROS was observed at 3 days post-TBI. Notably, 5-LOX inhibition with zileuton significantly reduced TBI-induced lipid ROS (*p* < 0.001; Figures 6A,C).

Subsequently, we used terminal deoxynucleotidyl transferase-mediated dUTP nick end labeling (TUNEL) immunofluorescence to assess neuronal cell apoptosis. Compared to the sham group, the expression of TUNEL-positive neuronal cells significantly increased at 3 days after injury, and zileuton administration partially suppressed neuronal cell apoptosis (*p* < 0.001; Figures 6B,D).

Previous studies have demonstrated that 5-LOX can peroxidize AA to form toxic phospholipid hydroperoxides, contributing to neuronal cell ferroptosis (Su et al., 2019). This led us to hypothesize that 5-LOX inhibition may also alleviate neuronal cell ferroptosis during TBI progression. As a result, we treated HT-22 neuronal cells with erastin and RSL3 to induce ferroptosis, and zileuton treatment significantly reduced both erastin- and RSL3-induced cell death (Supplementary Figure S2).

### LT-induced inflammatory pathway activation in BV2 microglia cells

**FIGURE 7:**
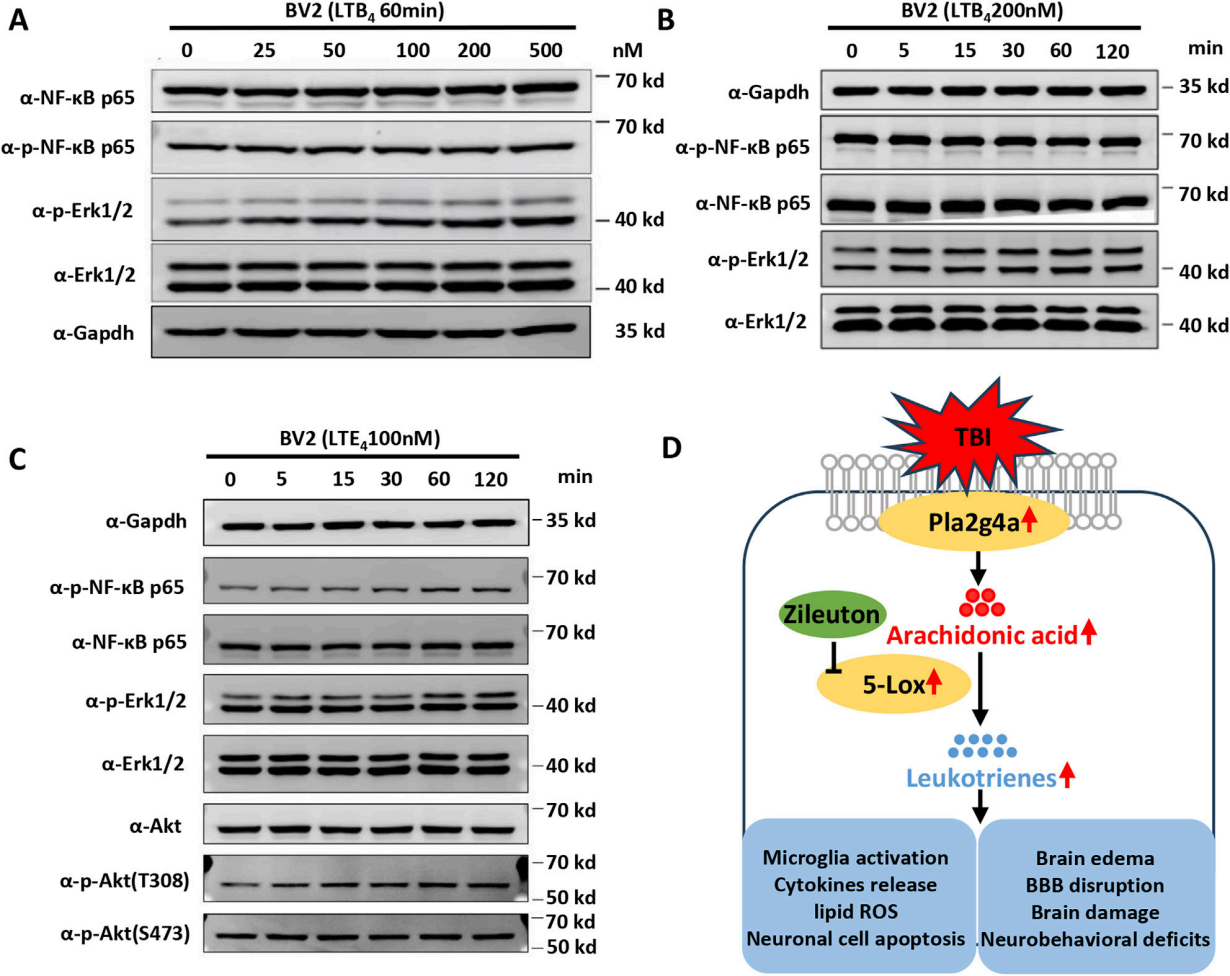
Leukotrienes (LTs) induced inflammatory pathway activation in BV2 microglial cells. **(A)** Representative dose-dependent and **(B)** time-dependent Western blot results for NF-κB p65, phospho-NF-κB p65, phospho-Erk1/2, and Erk1/2 expression in LTB4-treated BV2. **(C)** Representative Western blot results for NF-κB p65, phospho-NF-κB p65, phospho-Erk1/2, Erk1/2 Akt, phospho-Akt at T308, and phospho-Akt at S473 in LTE4-treated BV2. **(D)** Proposed model of the suppression of TBI-induced microglial activation, cytokine release, brain damage, and neurobehavioral deficits by zileuton through the inhibition of LT release.

AA is catalyzed by 5-LOX into LTA4, which can subsequently be converted to either LTB4 or LTC4, and then to LTD4 and LTE4. To explore the underlying signaling changes induced by LTs in microglial activation and the proinflammatory response after TBI, BV2 microglial cells were treated with LTB4 and LTE4. Western blot analysis was performed to examine the effects of LTB4 and LTE4 on the phosphorylation of ERK, NF-κB, and AKT *in vitro*. LTB4 significantly increased ERK phosphorylation in a time- and dose-dependent manner, without affecting the phosphorylation levels of NF-κB or AKT (Figures 7A, B). Meanwhile, LTE4 significantly increased the phosphorylation of ERK, NF-κB, and Akt at T308, but did not induce phosphorylation of Akt at S473 (Figure 7C). These findings suggest distinct roles for LTB4 and LTE4 in microglial activation, with LTB4 primarily influencing ERK phosphorylation and LTE4 affecting the phosphorylation of ERK, NF-κB, and Akt at T308. These data indicate that 5-LOX inhibition suppresses microglial activation by reducing LT release through inflammatory pathways (Figure 7D).

## Discussion

In the present study, we initially observed upregulation of the AA pathway, particularly the AA/5-LOX/LT axis, after TBI, suggesting a significant role for altered lipid metabolism in TBI processes. Subsequently, treatment with zileuton, a selective 5-LOX inhibitor, significantly ameliorated brain damage and neurobehavioral deficits by modulating the neuroinflammatory response following TBI. Furthermore, we found that zileuton inhibited the release of LTs, which activated inflammation-related pathways by phosphorylating ERK, NF-κB, and Akt. These results indicate that zileuton effectively inhibits 5-LOX activity and may be a promising treatment for TBI.

AA, released from inner membrane phospholipids by phospholipase enzymes, is metabolized through several pathways including the LOX, cytochrome P450 (CYP), and cyclooxygenase (COX) pathways, resulting in the production of 5-hydroxyeicosatetraenoic acids (HETEs), LTs, mid-chain HETEs, epoxyeicosatrienoic acid (EET) regioisomers, prostanoids, prostacyclin, and thromboxane. These AA-derived lipid mediators play crucial roles in initiating and resolving acute inflammatory processes. Previous studies have reported elevated levels of AA, and its metabolites, in the cerebrospinal fluid of TBI patients, correlating with adverse outcomes (Farias et al., 2011). The present study demonstrated cytoplasmic Pla2g4a upregulation and activation of the 5-LOX pathway of AA metabolism after TBI, particularly the upregulation of LTB4. These findings highlight the importance of AA metabolism through the 5-LOX pathway in the pathophysiological processes of TBI.

5-LOX is a key enzyme in the AA metabolic pathway, exhibiting widespread expression in both human and mouse brains. Previous studies have indicated its localization in neurons and glial cells, with upregulation observed during cerebral ischemic injury and neurodegenerative diseases in the human brain (Chen et al., 2020a). In specimens from TBI patients, 5-LOX was expressed in the cytosol and nucleus of neutrophils, neurons, GFAP-positive glial cells, and endothelial cells (Yan et al., 2021). Similarly, in the brains of mice with Alzheimer’s disease, 5-LOX was identified in both neurons and microglial cells (Michael et al., 2020). These diverse expression patterns highlight the complex roles of 5-LOX in the pathophysiology of certain neurological diseases, underscoring the need for a deeper understanding of its function in various cell types within the brain, particularly after TBI.

Our study revealed significant upregulation of 5-LOX expression in pericontusional brain tissue following TBI. Utilizing both single-cell RNA-seq analysis and immunofluorescence staining, we demonstrated the localization of 5-LOX to microglia, excluding neurons and GFAP-positive astrocytes. Moreover, we observed post-TBI upregulation of FLAP, a protein previously shown to be expressed exclusively in microglial cells in mouse brains (Michael et al., 2020). Considering the potential toxicity of AA and its role as an inducer of excessive ROS in microglia, we hypothesized that the coordinated action of 5-LOX with FLAP might be responsible for the excessive AA production in microglial cells following TBI. However, the regulatory mechanisms governing 5-LOX expression after TBI remain unclear. Notably, *in vitro* stimulation of BV2 microglia with lipopolysaccharide (LPS) did not induce 5-LOX upregulation, while both TNF-α and LPS stimulation of macrophages significantly upregulated 5-LOX expression both *in vitro* and *in vivo* (Rossi et al., 2010).

TBI resulted in increased release of LTB4 and cysteinyl LTs (LTC4, LTD4, and LTE4) (Farias et al., 2009). These lipid mediators play a crucial role in secondary injury processes, including increased blood–brain barrier permeability, inflammatory cell infiltration, the inflammatory response, and the formation of cerebral edema, all worsening neurological outcomes. The pivotal step in LT biosynthesis involves the coordinated action of 5-LOX and FLAP, converting AA to LTA4 (the precursor of LTs). Inhibition of LT formation by targeting FLAP has been demonstrated to significantly reduce brain lesion volume and ameliorate cognitive deficits in a fluid percussion injury model of TBI (Corser-Jensen et al., 2014). Consistent with the effects of FLAP inhibition, our study observed that 5-LOX inhibition with zileuton significantly reduced LTB4 formation, brain edema, and IgG extravasation, resulting in improved neurological outcomes.

The beneficial effects of zileuton administration and the cell type-specific upregulation of 5-LOX prompted exploration of the effects of 5-LOX inhibition on microglial activation and the inflammatory response after TBI. TBI triggered immune cell activation and inflammatory cytokine release, leading to progressive damage in areas close to the injury. Zileuton-related 5-LOX inhibition significantly reduced microglial activation, accompanied by the downregulation of genes involved in cytokine–cytokine receptor interaction and the chemokine signaling pathway. RNA-seq analysis identified several downregulated genes, including Il-1β, Ccl7, secreted phosphoprotein-1 (Spp1), chemokine receptor (Ccr1), Ccl2, and Il-10. These genes play an important role in the inflammatory reactions seen in neurological diseases. The inflammatory cytokines Il-1β, Ccl7, Spp1, Ccr1, Ccl2, and Il-10 promoted microglial and macrophage infiltration in the pericontusional area after TBI. Manipulation of these cytokines through genetic or pharmacological approaches has been shown to significantly reduce inflammatory cell accumulation and alleviate brain damage compared to control mice with TBI (Cherry et al., 2020; Xue et al., 2021; Flygt et al., 2018; Sugiyama et al., 2019; Gu et al., 2022; Semple et al., 2010). Additionally, antagonizing Ccr1 activity has been demonstrated to protect the hemorrhagic brain from inflammatory injury (Yan et al., 2020). TBI induces inflammatory cell infiltration, increased cytokine release, and microglial activation in pericontusional brain tissue, creating a vicious cycle that often culminates in excessive cerebral inflammation. Our data indicate that 5-LOX inhibition interrupts this cycle, at least partly, by reducing LT release. The alleviation of neuroinflammation by zileuton also reduces secondary brain injury, including lipid ROS generation and neuronal cell apoptosis, resulting in improved neurobehavioral recovery after TBI.

The anti-inflammatory effects of 5-LOX inhibition have also been demonstrated in other neuropsychiatric disorders. In a model of depression, zileuton significantly improved depressive‐like behavior by suppressing hippocampal TNF α, IL-1β, nuclear NF-κB p65 and microglial activation, reducing neuronal apoptosis and restoring synaptic proteins and neurogenesis (Liu et al., 2020). In a rat model of middle cerebral artery occlusion, zileuton reduced infarct volume, neurological deficit scores, cerebral water content, ischemic neuronal injury, downregulated NF-κB p65, COX 2 and TNF-α release (Tu et al., 2016). In a model of retinal degeneration, manipulation of 5-LOX activity attenuated ROS-induced lipid peroxidation, mitochondrial damage, DNA damage and ferroptotic cell death, preserving photoreceptor integrity and reducing retinal inflammation and degeneration (Lee et al., 2022). Persistent 5-LOX activation is emerging as a hallmark of chronic neurological conditions. In models of Gulf War Illness, 5-LOX and leukotriene expression remained elevated for 6 months after exposure, correlating with sustained microglial activation and spatial memory deficits (Attaluri et al., 2022). Zileuton can prevent inflammation in the chronic stage of both Gulf War Illness and cardiac Chagas disease (Attaluri et al., 2022; Giannopoulos et al., 2019). By concurrently modulating immune cell activation, cytokine release, and oxidative damage pathways, zileuton treatment addresses multiple nodes in the neuroinflammatory cascade. The conserved anti-inflammatory profile across these models positions 5-LOX inhibition as a promising translational strategy for the treatment of TBI to improve neurological outcomes. The demonstrated efficacy in both acute and chronic phases warrants further investigation into optimized dosing regimens and combination therapies.

Beneficial effects of zileuton administration, particularly in reducing LT biosynthesis, have been observed in various central nervous system disorders (Michael et al., 2020; Chen et al., 2020a; Giannopoulos et al., 2019). Zileuton treatment has demonstrated positive outcomes in intracerebral hemorrhage models, where it decreased neutrophil infiltration and attenuated microglial activation through the LTB4-BLT1 signaling axis, leading to improved sensorimotor function recovery (Hijioka et al., 2017; Hijioka et al., 2020). Moreover, 5-LOX inhibition has shown efficacy in reducing proinflammatory cytokines, preventing nuclear translocation and activation of NF-κB in rat models of cerebral ischemic injury (Tu et al., 2010; Chen et al., 2020b) and reducing TNF-α release in ischemic brain injury in a PI3K/Akt-dependent manner (Tu et al., 2016). However, the mechanisms underlying zileuton’s therapeutic effects and reduced LT biosynthesis in microglia and infiltrating immune cells in TBI have not been widely investigated. Stimulation of BV2 microglia with LTB4 or LTE4 had differential effects on microglial activation, with LTB4 increasing ERK phosphorylation and LTE4 increasing the phosphorylation of ERK, NF-κB, and Akt at T308. This indicates complex and synergistic effects of LTs in the inflammatory processes associated with TBI, which involve immune cell infiltration and microglial activation (Alam et al., 2020; Bolte and Lukens, 2021; Dean et al., 2024). Taken together, these observations suggest that 5-LOX inhibition reduces LT products and subsequent microglial activation via inflammatory pathways, including the ERK, NF-κB, and Akt pathways. Further studies are needed to investigate the influence of each LT on different immune cell types, including microglia, neutrophils, monocytes, and macrophages, to unravel the temporal and spatial immune responses following TBI (Chen and Zou, 2022).

This study has several limitations. First, we have identified specific effects and mechanisms of 5-LOX and LTs in the early phase after TBI, the subsequent influence in the long-term phase needs further investigation. Second, although the anti-inflammatory effect of Zileuton is a very effective strategy in the treatment of TBI, it is important to remember that inflammation also plays a critical role in the regeneration and repair of brain tissue (Cooke, 2019). Therefore, while zileuton is a promising anti-inflammatory agent, it is important to balance its efficacy in limiting harmful inflammation with its potential effects on regenerative processes. Thirdly, specific pathogen-free (SPF) environments do not fully replicate conditions of natural exposure (Beura et al., 2016). Therefore, results should be cautiously extrapolated to pathogen-exposed animal models or humans.

## Conclusion

In conclusion, AA biosynthesis and subsequent metabolism via 5-LOX, which releases LTs, are upregulated after TBI. The exclusive expression of 5-LOX in microglial cells, and its correlation with the inflammatory response, suggest that activated microglia are the cellular source of LTs in the brain. LTs may stimulate microglia in an autocrine or paracrine manner through inflammatory pathways, including the ERK, NF-κB, and Akt pathways. Inhibition of 5-LOX with zileuton significantly reduced LTB4 formation and ameliorated neuroinflammation-related secondary brain damage, resulting in improved neurological recovery. These findings improve our understanding of the cellular expression of 5-LOX in the brain and highlight its potential as a therapeutic target for TBI treatment.

## Data Availability

The original contributions presented in the study are publicly available. This data can be found here: GEO repository, accession number GSE297412 (https://www.ncbi.nlm.nih.gov/geo/query/acc.cgi?acc=GSE297412).

## Supplementary material

SUPPLEMENTARY ADDITIONAL FILE 1 Kyoto Encyclopedia of Genes and Genomes (KEGG) enriched pathways at 7 days after TBI without treatment.

SUPPLEMENTARY ADDITIONAL FILE 2 KEGG enriched pathways at 7 days after TBI with zileuton treatment.

SUPPLEMENTARY ADDITIONAL FILE 3 The primer pair sequences used for RT-PCR reactions.

SUPPLEMENTARY FIGURE S1 mRNA levels of TNF-α, Il-17, Ccl12, and Il-6 were measured in the vehicle- and zileuton-treated mice at 3 days after TBI. Zileuton did not reduce TBI induced TNF-α, Il-17, Ccl12, and Il-6 upregulation.

SUPPLEMENTARY FIGURE S2 Dose dependent cell viability in Erastin- and RSL3-induced cell death with or without zileuton treatment. Zileuton inhibit erastin- and RSL3-induced HT-22 cell death.

SUPPLEMENTARY FIGURE S3 Changes in AA, prostaglandin E2 and lipoxin A4 at 1, 3 and 7 days after TBI. AA, Prostaglandin E2 and Lipoxin A4 concentrations in brain tissue were measured in sham and TBI mice at 1, 3 and 7 days after TBI (n = 10, = 12, and = 11 for the sham, 1-day, 3-day and 7-day groups, respectively, *p < 0.05).

## Abbreviations

TBI, Traumatic brain injury; 5-LOX, 5-lipoxygenase; LT, Leukotriene; CCI, Controlled cortical impact; RT-PCR, Real-time polymerase chain reaction; KEGG, Kyoto Encyclopedia of Genes and Genomes (KEGG); LC-MS/MS, Liquid chromatography mass spectrometry/mass spectrometry; ELISA, Enzyme linked immunosorbent assay (); BBB, Blood brain barrier; AA, Arachidonic acid; Pla2g4a, Phospholipase A2-IVA; Il-1β, Interleukin 1 beta; Ccl7 C-C Motif Chemokine Ligand 7; Spp1, Secreted Phosphoprotein 1; Ccr1 C-C, Motif Chemokine Receptor 1; Ccl2 C-C, Motif Chemokine Ligand 2; Il-10, Interleukin 10; ROS, Reactive oxygen species; ERK, Extracellular signal-regulated kinase; NF-κb, Nuclear factor-κb.
