# Supplementary figures and images for: Zileuton protects against arachidonic acid/5-lipoxygenase/leukotriene axis-mediated neuroinflammation in experimental traumatic brain injury
Source: Front Pharmacol. 2025 Jun 5;16:1516836. doi: 10.3389/fphar.2025.1516836 (PMC12176805; doi:10.3389/fphar.2025.1516836)

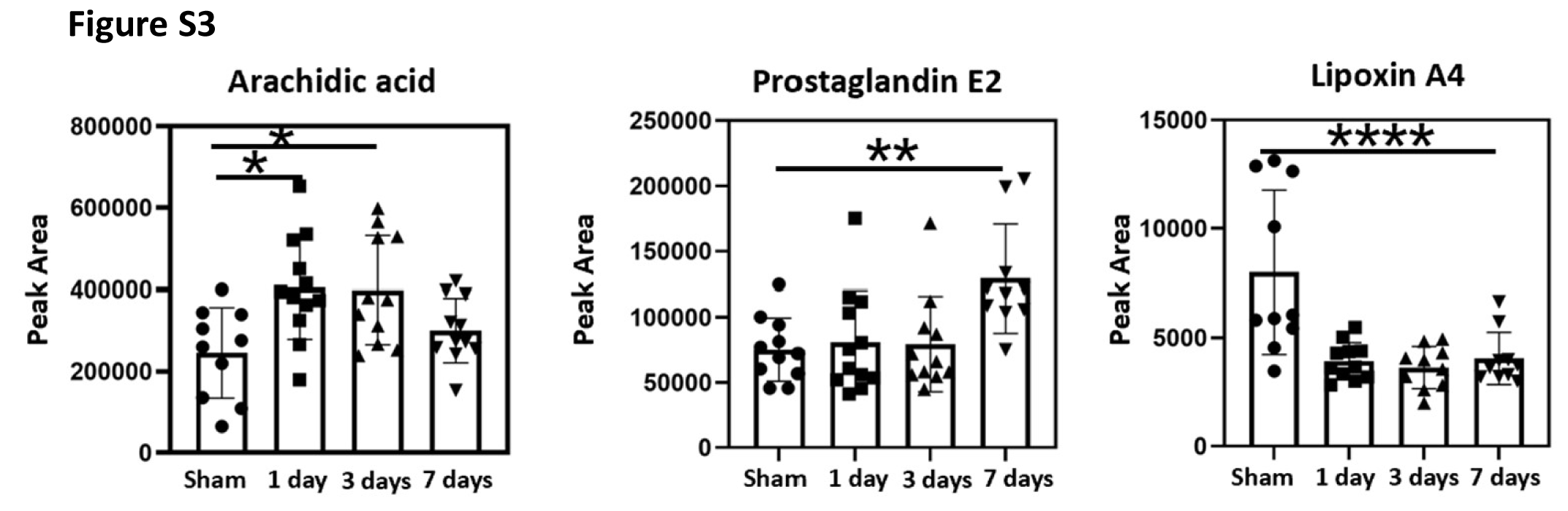

Supplement: Supplementary file 1 [file Image3.tif]

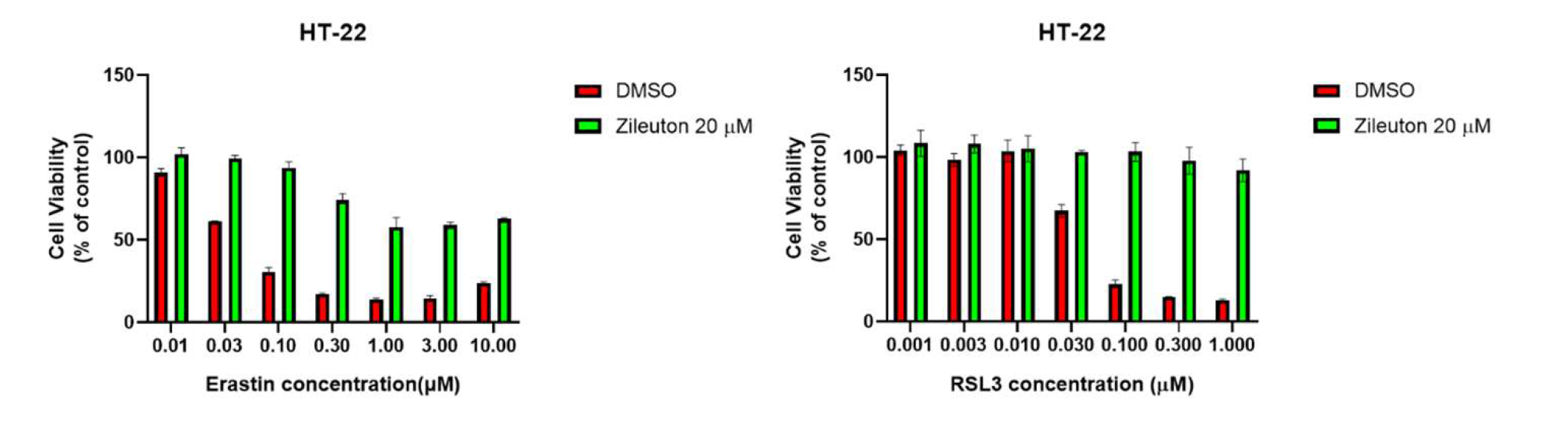

Supplement: Supplementary file 2 [file Image2.tif]

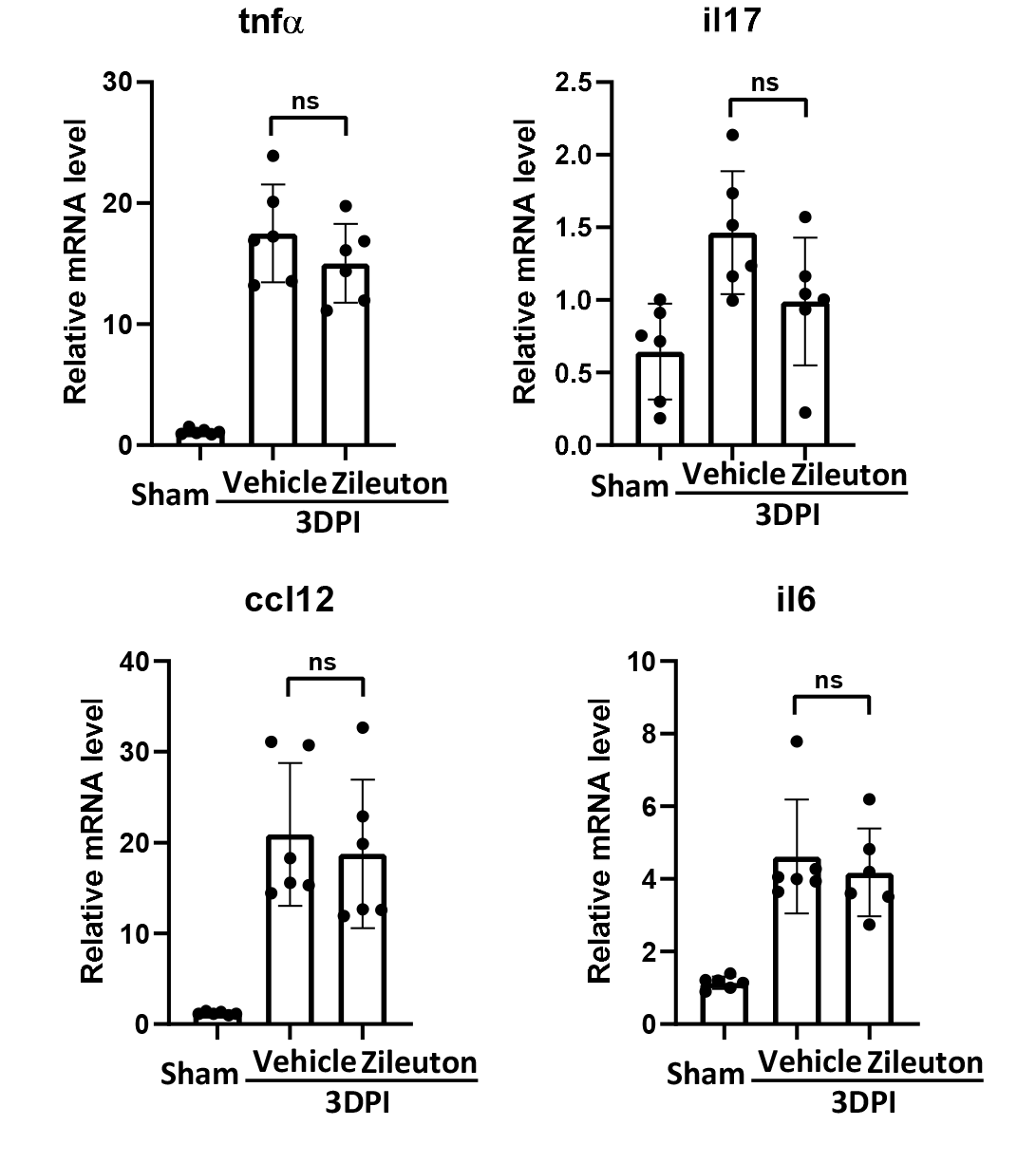

Supplement: Supplementary file 3 [file Image1.tif]
